# HIV-1 Nef Employs Two Distinct Mechanisms to Modulate Lck Subcellular Localization and TCR Induced Actin Remodeling

**DOI:** 10.1371/journal.pone.0001212

**Published:** 2007-11-21

**Authors:** Claudia Haller, Susanne Rauch, Oliver T. Fackler

**Affiliations:** Abteilung Virologie, Universitätsklinikum Heidelberg, Heidelberg, Germany; Karolinska Institutet, Sweden

## Abstract

The Nef protein acts as critical factor during HIV pathogenesis by increasing HIV replication *in vivo* via the modulation of host cell vesicle transport and signal transduction processes. Recent studies suggested that Nef alters formation and function of immunological synapses (IS), thereby modulating exogenous T-cell receptor (TCR) stimulation to balance between partial T cell activation required for HIV-1 spread and prevention of activation induced cell death. Alterations of IS function by Nef include interference with cell spreading and actin polymerization upon TCR engagement, a pronounced intracellular accumulation of the Src kinase Lck and its reduced IS recruitment. Here we use a combination of Nef mutagenesis and pharmacological inhibition to analyze the relative contribution of these effects to Nef mediated alterations of IS organization and function on TCR stimulatory surfaces. Inhibition of actin polymerization and IS recruitment of Lck were governed by identical Nef determinants and correlated well with Nef's association with Pak2 kinase activity. In contrast, Nef mediated Lck endosomal accumulation was separable from these effects, occurred independently of Pak2, required integrity of the microtubule rather than the actin filament system and thus represents a distinct Nef activity. Finally, reduction of TCR signal transmission by Nef was linked to altered actin remodeling and Lck IS recruitment but did not require endosomal Lck rerouting. Thus, Nef affects IS function via multiple independent mechanisms to optimize virus replication in the infected host.

## Introduction

The activation state of target cells often dictates the efficacy by which virus pathogens spread in the infected host. In the case of Human Immunodeficiency Virus (HIV), quiescent T lymphocytes, besides macrophages the main target cell population in the human body, are readily infected by the virus, however do not support efficient replication due to multiple early post entry replication blocks [1]. Partial T cell activation relieves these barriers allowing for sustained virus propagation. Host cell death, induced by expression of viral gene products or hyperactivation of the cells, imposes an additional limitation to virus spread [2]. Evidence has recently accumulated that the viral protein Nef plays a critical role in balancing the requirements of cell activation and apoptosis prevention [3]. Nef is an accessory gene product unique to the primate lentiviruses HIV-1, HIV-2 and Simian Immunodeficiency virus (SIV). Long recognized as essential for efficient virus spread and thus disease progression in the infected host [4], [5], [6], the underlying molecular mechanisms have remained largely unexplained. A large body of evidence suggests that Nef profoundly manipulates HIV target cells by altering a variety of signal transduction and protein sorting processes. With respect to T lymphocyte infection, Nef is known to affect T cell receptor (TCR) signaling transduction. Generally Nef is viewed as an enhancer of endogenous TCR signaling that can sensitize isolated T cells for activation [7], [8], [9], [10], [11], [12], thereby increasing their permissivity to HIV infection. Several reports now indicate that, in the context of exogenous TCR activation, Nef expression causes a reduction of signal transmission to prevent cell hyperactivation to prolong the lifespan of infected cells [13], [14], [15].

Physiologically, exogenous T cell activation occurs in the context of a close contact between an antigen presenting cell (APC) and a T cell, referred to as the immunological synapse (IS). Engagement of the TCR by MHC-I bound antigenic peptides triggers profound reorganization of the protein and lipid composition at the IS, leading to the formation of signaling competent protein microclusters [16], [17]. Lateral sorting processes at the IS as well as the stabilization of cell-cell contacts are mediated by massive actin rearrangements and TCR induced actin remodeling is a prerequisite for subsequent signaling [18], [19]. IS composition and function is also controlled by microtubule mediated transport of signaling components and secretory cargo, which often involves reorientation of the microtubule organizing centre towards the synapse [18]. Finally, removal of cell surface receptors such as the TCR via endocytic transport contributes in various ways to IS function: internalization and subsequent recruitment to IS ensures high local concentrations at the IS while TCR internalization can also lead to lysosomal degradation and thus termination of signaling [20]. Successful TCR signal initiation induces a characteristic cascade of tyrosine phosphorylation events, Ca2^+^ release and the activation of transcription factors such as NF-AT that regulate gene expression of e.g. IL-2 [21].

Several independent studies recently reported that Nef potently affects formation and function of the IS. Using APC-T cell conjugates or TCR stimulatory surfaces, the overall effect of Nef was to reduce but not abrogate the TCR signal in the recipient T lymphocyte , resulting in a significant reduction of TCR induced tyrosine phosphorylation [13], [15]. As analyzed in virally infected T lymphocytes, this also resulted in a negative effect of Nef on IL-2 production [15]. Several effects of Nef expression were observed that might explain these effects on IS function. First, Nef markedly reduced actin polymerization and cell spreading induced upon contact of T lymphocytes with TCR stimulatory surfaces [13]. Second, Nef caused the pronounced steady state accumulation of the TCR proximal Src kinase Lck as well as of TCR-CD3 in an intracellular endosomal compartment [15]. Third, Nef also prevented efficient recycling of these two components towards the IS following TCR engagement [15]. These studies thus revealed profound effects of Nef on intracellular sorting and actin remodeling at the IS. How these individual effects on IS organization are mediated by Nef, whether they represent independent or interconnected activities and how they each contribute to Nef mediated alterations in IS function, however remained unclear. In this study, we therefore employed a combination of Nef mutagenesis and pharmacological inhibition to address these questions. We find that the effects of Nef on actin remodeling and Lck IS recruitment are genetically linked, correlate with Nef's ability to associate with the cellular Pak2 kinase and determine the observed reduction in TCR signal transmission. In contrast, intracellular accumulation of Lck was mediated by an independent Nef activity, involved the microtubule network and was insufficient for the modulation of the tested aspects of TCR signaling. Nef therefore employs multiple independent mechanisms to affect IS architecture.

## Results

### Nef triggers intracellular accumulation of Lck and interferes with its recruitment to sites of TCR engagement

**Figure 1:**
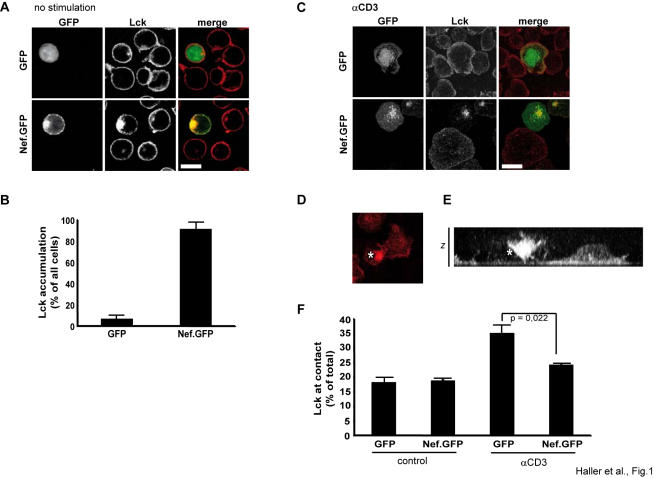
HIV-1 Nef triggers intracellular accumulation of Lck and prevents Lck recruitment to sites of T cell receptor engagement. (A) Confocal microscopy analysis of Jurkat T lymphocytes transfected with expression plasmids for GFP or Nef.GFP after staining for endogenous Lck. Depicted is a representative confocal section through the middle of the cell. White bar = 10 µM. (B) Quantification of cells displaying pronounced intracellular Lck accumulation. Values are the arithmetic means of at least three independent experiments+s.d. in which over 100 cells were counted per condition. (C) Confocal microscopy analysis of Jurkat T lymphocytes transfected with expression plasmids for GFP or Nef.GFP after 5min incubation on anti-CD3 coated coverglasses and subsequent staining for endogenous Lck. Cells were analyzed by confocal microscopy and representative z-sections directly above the coverglass are presented. White bar = 10 µM. (D) Projection of a z-stack series of confocal sections of Jurkat T lymphocytes on stimulatory coverglasses after staining for Lck. The asterisk denotes a Nef.GFP expressing cell. (E) Side projection of a confocal z-stack series of the cells depicted in D. (F) Quantification of the pixel intensity of the Lck signal at the z-section near the coverglass surface relative to the total pixel intensity in all z-sections from the same cell. Values are the arithmetic means of at least three independent experiments+s.e.m. in which over 10 randomly selected cells were analyzed per condition. Control, incubation of cells on non-stimulatory coverglasses; αCD3, incubation of cells on anti-CD3 coated coverglasses.

Nef has recently been suggested to interfere with the intracellular sorting of the TCR proximal kinase Lck in HIV-1 infected primary human T lymphocytes [15]. To verify this finding, Jurkat T lymphocytes were transiently transfected with expression plasmids for GFP (GFP) or a GFP fusion protein of Nef from HIV-1 SF2 (Nef.GFP) that is functionally analogous to non fusion Nef [11], [22] and the intracellulular localization of endogenous Lck was analyzed by confocal microscopy (Fig. 1A). Expectedly, Lck was most prominent at the plasma membrane with some additional localization to an intracellular compartment in the presence of GFP or in non-transfected cells. In contrast, expression of Nef.GFP strongly increased the localization of Lck to this intracellular compartment causing reduced Lck levels at the plasma membrane. Partial colocalization of this compartment with transferrin receptor, Rab4 and Rab11 (data not shown) are in line with the suggested identification of the compartment as recycling endosomes (RE) [15]. Quantification of Lck localization revealed that Nef caused a more than 9-fold increase in the percentage of cells displaying pronounced intracellular Lck accumulation (Fig. 1B).

Thoulouze et al. also reported that in addition to changing the steady state localization of Lck, Nef impairs the recruitment of Lck to the IS following TCR engagement [15]. We previously established TCR stimulatory coverglasses coated with anti-CD3 antibodies as experimental system to study Nef's effects on TCR induced actin dynamics [13]. Such stimulatory surfaces are widely used to mimic the formation of IS between APCs and T lymphocytes and to study TCR induced actin dynamics as well as the motility of signaling competent microcluster. Importantly, key features of TCR signaling events such as induction of tyrosine phosphorylation and Ca2+ release are faithfully preserved in this experimental system [23], [24], [25]. We therefore tested if we were able to detect the described inhibition of IS recruitment of Lck by Nef using this approach and analyzed the distribution of endogenous Lck in GFP or Nef.GFP expressing Jukat T lymphocytes following 5min incubation on TCR stimulatory coverglasses (Fig. 1C). As reported previously [13], Nef.GFP potently interfered with cell spreading observed in GFP expressing control cells. Moreover, the pronounced intracellular Lck accumulation was preserved in Nef.GFP expressing cells following CD3 stimulation. To quantify the recruitment of Lck to the stimulatory contact, confocal z-stacks spanning from top to bottom of the cells were recorded (Fig. 1D). A side projection of such a 3D stack reveals the prominent intracellular Lck accumulation in the Nef.GFP expressing cell (Fig. 1E). On several occasions, we observed a thin connection between the intracellular compartment and the stimulatory contact as shown in Fig. 1E. In contrast, Lck appears markedly enriched at the stimulatory contact in the adjacent Nef negative cell. To quantify the effects of Nef on the distribution of Lck, overall Lck pixel intensities and their relative accumulation at the contact site were determined from these stacks of confocal sections (Fig. 1F). This analysis revealed that upon TCR engagement, significant amounts of Lck are recruited to the contacts in GFP expressing control cells. In contrast, this process is significantly inhibited in Nef.GFP expressing cells in which Lck levels at the contact sites are only slightly elevated following TCR stimulation. Analyses on TCR stimulatory surfaces thus reflect the Nef mediated inhibition of Lck recruitment to the IS.

### Lck accumulation is a conserved activity of lentiviral Nef proteins

**Figure 2:**
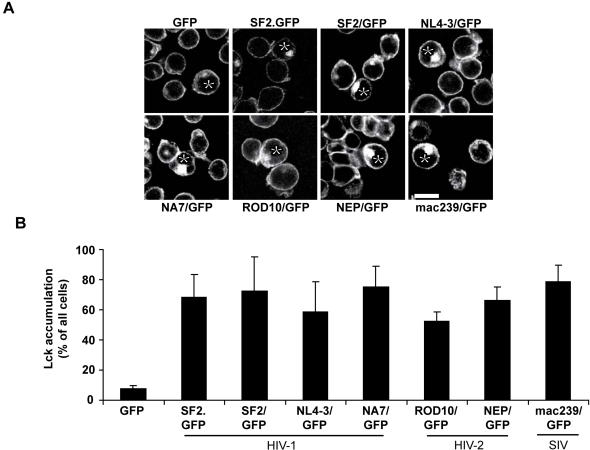
Intracellular Lck accumulation is a conserved activity of lentiviral Nef proteins. (A) Confocal microscopy analysis of Jurkat T lymphocytes transfected with the indicated expression plasmids after staining for endogenous Lck. Depicted is a representative confocal section through the middle of the cell. Asterisks denote GFP positive cells. White bar = 10 µM. (B) Quantification of the experiment shown in A. Values are the arithmetic means of at least three independent experiments+s.d. in which over 100 cells were counted per condition.

Due to the sequence variability among Nef variants and the plethora of described Nef activities, we next asked whether the ability to cause intracellular Lck accumulation is a conserved activity. As depicted in Fig. 2A, Nef proteins from various HIV-1 as well as HIV-2 isolates but also from SIV mac239 readily triggered the enrichment of intracellular Lck. All Nef proteins analyzed exerted this effect with comparable efficiency and irrespective of whether expressed as a GFP fusion protein or from a bicistronic expression plasmid (e.g. SF2/GFP). We concluded that targeting Lck to a RE-like compartment is a conserved activity of lentiviral Nef proteins.

### Mapping of determinants in Nef for Lck accumulation

**Figure 3:**
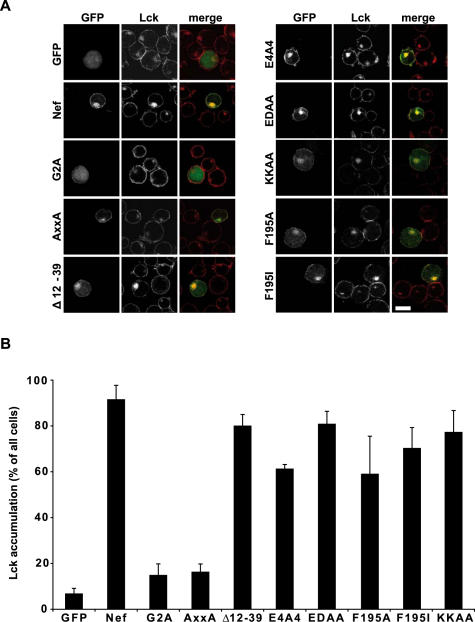
Intracellular Lck accumulation requires membrane attachment and the SH3 binding motif of Nef. (A) Confocal microscopy analysis of Jurkat T lymphocytes transfected with the indicated expression plasmids after staining for endogenous Lck. Depicted is a representative confocal section through the middle of the cell. White bar = 10 µM. (B) Quantification of the experiment shown in A. Values are the arithmetic means of at least three independent experiments+s.d. in which over 100 cells were counted per condition.

We next made use of a panel of characterized Nef mutants to define the determinants that govern its effects on subcellular Lck distribution (Fig. 3). One set of Nef mutants displayed essentially wild type activity in accumulating Lck. This included mutants deficient in interacting with the Nef associated kinase complex (Δ12-39) [7], the PACS sorting adaptor (E4A4) [26], endocytosis adaptor complexes (EDAA) [27], the Pak2 kinase (F195A, F195I) [28] or the incorporation into detergent-resistant membrane microdomains (DRM) (KKAA) [29]. In contrast, the presence of the G2A and AxxA Nef mutants caused virtually no change in the subcellular localization of Lck. The G2A mutation prevents the N-terminal myristoylation of Nef to disrupt most of its membrane association which is required for many Nef activities. The AxxA mutation interferes with the interaction of Nef with SH3 domain containing host cell proteins that have been implied in several Nef activities including host cell signal transduction and sorting processes [30], [31]. Lck accumulation by Nef thus likely involves membrane association and SH3 interactions of the viral protein.

### Discordance between Nef determinants for actin ring inhibition and its influence on Lck contact site recruitment versus intracellular Lck accumulation

**Figure 4:**
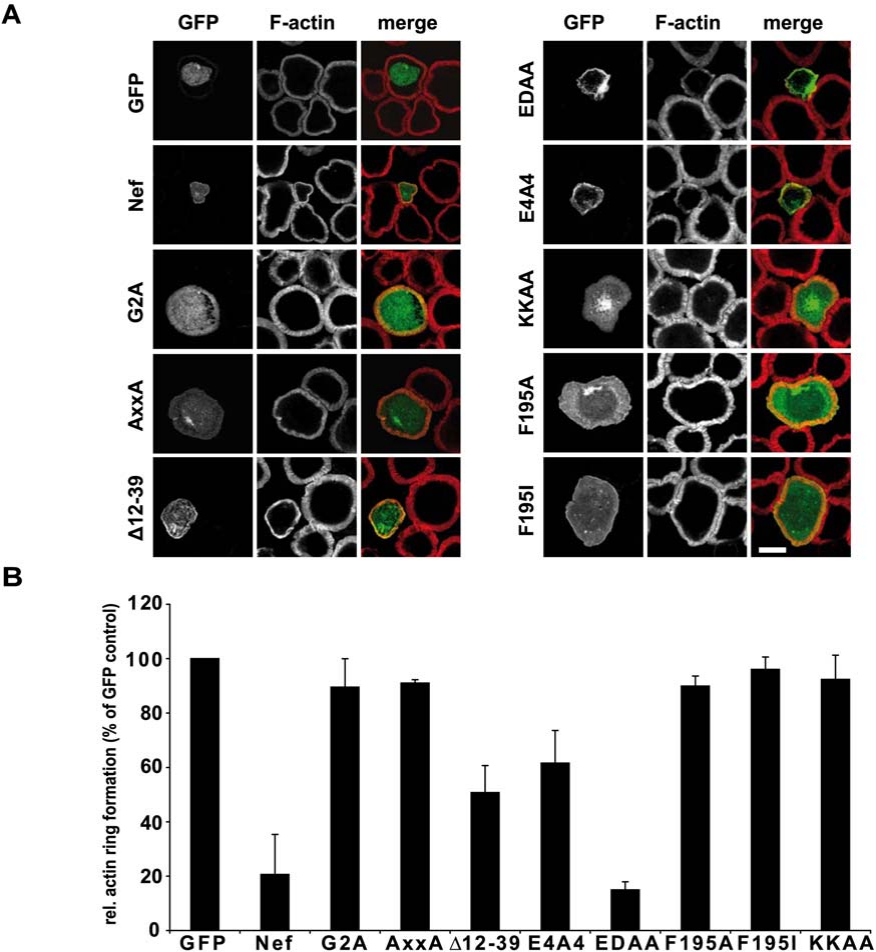
Inhibition of TCR induced actin remodeling depends on the protein interaction motif surrounding F195 in Nef. (A) Confocal microscopy analysis of Jurkat T lymphocytes transfected with the indicated expression plasmids after 5min incubation on anti-CD3 coated coverglasses and subsequent staining for F-actin. Depicted is a representative confocal section near the coverglass. White bar = 10 µM. (B) Quantification of the experiment shown in A. Shown is the percentage of cells exhibiting pronounced actin ring formation relative to the mean value of GFP expressing control cells that was arbitrarily set to 100%. Values are the arithmetic means of at least three independent experiments+s.d. in which over 100 cells were counted per condition.

**Figure 5:**
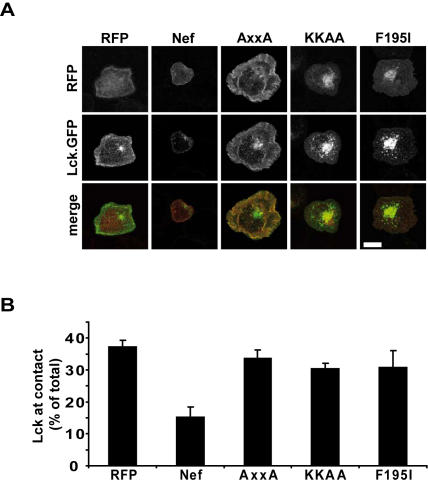
Intracellular Lck accumulation does not determine its recruitment to TCR contacts. (A) Confocal microscopy analysis of Jurkat T lymphocytes transfected with expression plasmids for the indicated RFP or Nef.RFP proteins as well as for Lck.GFP after 5min incubation on anti-CD3 coated coverglasses. Depicted is a representative confocal section near the coverglass. White bar = 10 µM. (B) Quantification of the experiment shown in A. Shown is the relative pixel intensity of the Lck.GFP signal at the z-section near the coverglass surface relative to the total pixel intensity in all z-sections from the same cell. Values are the arithmetic means of at least three independent experiments+s.e.m. in which over 10 randomly selected cells were investigated per condition.

With the intracellular accumulation of Lck, the prevention of its recruitment to sites of TCR engagement and the inhibition of actin remodeling, at least three responses to stimulation are affected by Nef early following activation of T lymphocytes. To address whether these effects are interdependent, we next defined the determinants for actin ring inhibition by Nef using the Nef mutant panel. Transfected Jurkat T lymphocytes were incubated for 5min on TCR stimulatory surfaces and stained for F-actin. While control cells efficiently spread and displayed the characteristic prominent circumferential actin rings, Nef.GFP potently prevented cell spreading and the polymerization of F-actin into thick rings with filopodia like protrusions (Fig. 4A and [13]). As reported previously [13], the EDAA Nef mutant was fully active and Δ12-39 and E4A4 displayed intermediate activity while G2A and AxxA Nef mutants were defective for this activity. Importantly, mutations in residue F195 as well as disruption of the DRM targeting motif KK completely abolished Nef's ability to interfere with TCR induced actin remodeling. These results define the F195 protein interaction surface as well as DRM incorporation as selective determinants for Nef's effect on actin dynamics but not on Lck accumulation. These two mutants together with the AxxA Nef mutant, which neither affects actin dynamics nor Lck localization, were therefore tested as mRFP1 fusion proteins for their ability to interfere with TCR contact site recruitment of Lck.GFP (Fig. 5). Also in this experimental setting, Nef.RFP caused a more than two-fold reduction in Lck recruitment towards the TCR stimulatory surface. Surprisingly however, all three Nef mutants analyzed did not exert any effect on the relocalization of Lck following TCR engagement. Thus, Lck accumulation and interference with TCR induced actin dynamics by Nef can be separated. Moreover, reduced Lck contact site recruitment correlates with Nef's effects on actin dynamics rather than with steady state Lck subcellular localization.

### Determinants for Nef's association with Pak2 activity

**Figure 6:**
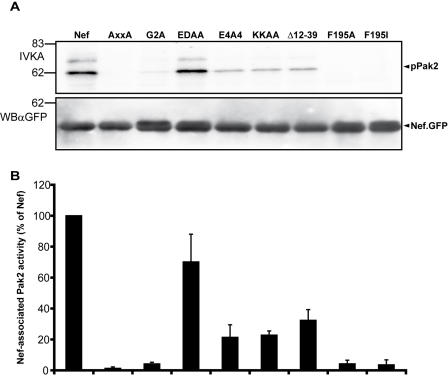
Analysis of Nef determinants for its association with cellular Pak2 activity. (A) Analysis of Pak2 association for various Nef mutants. Jurkat T lymphocytes were transfected with the indicated Nef.GFP expression plasmids and an *in vitro* kinase assay (IVKA) following anti-GFP immunoprecipitation was performed. Nef associated Pak2 activity is revealed by the phosphorylated 62 kDa band (IVKA, pPak2). WB αGFP depicts a western blot monitoring the amounts of Nef.GFP present in the IVKA reaction. (B) Quantification of the Nef associated Pak2 activity. Intensities of autophosphorylated Pak2 signals were quantified relative to the amounts of immunoisolated Nef.GFP. The relative associated Pak activity for Nef.GFP was arbitrarily set to 100%. Data are mean±s.d. of at least 5 independent experiments.

We previously reported that the inhibition of TCR induced actin ring formation might involve Nef's association with the activity of the cellular Pak2 kinase, a key regulator of cellular actin dynamics [13]. To address a potential role of this association for all Nef activities at the IS, we tested the Nef mutant panel for association with Pak2 kinase activity (Fig. 6). *In vitro* kinase assays (IVKA) were performed after immunoprecipitation of the various Nef.GFP proteins from Jurkat T lymphocytes, resulting in the autophosphorylation of Pak2 (62kDa) and a yet uncharacterized Pak2 substrate (approx. 70kDa) (Fig. 6A, upper panel). Amounts of phosphorylated Pak2 were normalized to the amounts of the respective Nef.GFP fusion protein present in the reaction (Fig. 6A, lower panel) to calculate the relative Nef associated Pak2 activity (Fig. 6B). As expected [32], [33], the ED motif was dispensable while the AxxA and G2A mutations almost completely abrogated the Nef-Pak2 association. Confirming recent findings [28], mutations at position F195 also potently blocked the Nef-Pak2 association. The E4A4, Δ12-39 and KKAA Nef mutants displayed significantly reduced activities relative to wt Nef, however retained detectable Pak2 association. These results revealed a good correlation between the degree of actin remodeling inhibition of this Nef mutant panel and its association with Pak2 activity.

### Different involvement of actin and microtubules in Nef effects at stimulatory contacts

**Figure 7:**
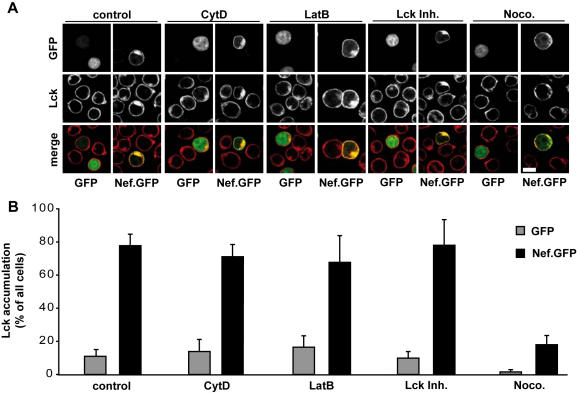
Sustained Nef induced Lck accumulation requires microtubule integrity. (A) Confocal microscopy analysis of Jurkat T lymphocytes transfected with GFP or Nef.GFP expression plasmids and stained for endogenous Lck after 30min incubation with the indicated drugs (CytD 5 µM; LatB 0.5 µM; Lck Inh. 3 µM; Noco. 10 µM; control, solvent control). Depicted is a representative confocal section through the middle of the cell. White bar = 10 µM. (B) Quantification of the experiment shown in A. Values are the arithmetic means of at least three independent experiments+s.d. in which over 100 cells were counted per condition.

**Figure 8:**
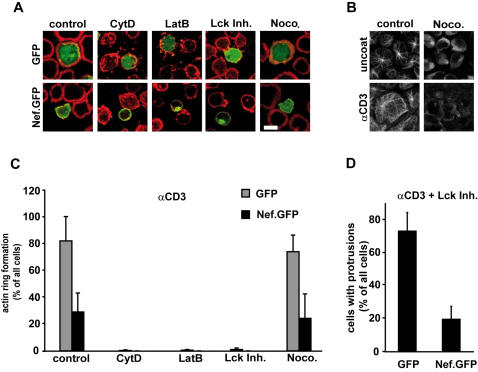
Effects of Nef on TCR induced actin dynamics are microtubule independent. (A) Confocal microscopy analysis of Jurkat T lymphocytes transfected with GFP or Nef.GFP expression plasmids after 5min incubation on anti-CD3 coated coverglasses and subsequent staining for F-actin (red). Prior to incubation with TCR stimulatory surfaces, cells were incubated for 30min with the indicated drugs (CytoD 5 µM, LatB 0.5 µM, Lck Inh. 3 µM, Noco. 10 µM). Depicted is the merge picture of a representative confocal section near the coverglass. White bar = 10 µM. (B) Confocal microscopy analysis of microtubules in Jurkat cells after 5min incubation on uncoated or CD3 stimulatory coverglasses (αCD3), respectively. Cells depicted on the right were incubated for 30min with 10 µM nocodazole. (C) Quantification of actin ring formation of the cells shown in A. Values are the arithmetic means of at least three independent experiments+s.d. in which over 100 cells were counted per condition. (D) Quantification of cells carrying actin rich protrusions after anti-CD3 stimulation in the presence of Lck inhibitor.

The above results suggested that Lck accumulation and inhibition of TCR induced actin dynamics may be distinct activities of Nef. To test this hypothesis further we investigated the role of actin and microtubule filament systems in both processes by applying specific drugs that depolymerize actin filaments (cytochalasin D (CytD) and latrunculinB (LatB)) or microtubules (nocodazole/Noco.), respectively (Fig. 7). To test for the role of Lck kinase activity, a specific kinase inhibitor (Lck Inh.) was used. All drugs did not affect cell viability at the concentrations used as their effects were fully reversed 4 hours following washout of the drug (data not shown). The efficacy of the cytoskeleton depolymerizing drugs is shown in Fig. 8 (see below). Addition of the Lck inhibitor efficiently prevented the induction of Lck specific tyrosine phosphorylation in T lymphocytes following TCR stimulation (data not shown). We first assessed the effect of these drugs on Nef mediated intracellular targeting of Lck. Depolymerization of F-actin for 30min had no marked qualitative (Fig. 7A) or quantitative (Fig. 7B) effects on Nef's ability to accumulate Lck in the RE-like compartment. Interestingly, similar results were obtained upon inhibition of Lck activity. More extended drug treatment for up to 2 hours did not alter the outcome of these experiments (data not shown). In contrast, the intracellular accumulation of Lck was profoundly disturbed upon depolymerization of microtubules for 30 min, thereby almost reversing the Nef induced accumulation. The residual localization of Lck to the RE-like compartment in the absence of Nef was also disrupted upon nocodazole treatment. These results demonstrate that the integrity of the intracellular compartment in which Lck accumulates in the presence of Nef is nocodazole sensitive but resistant to F-actin depolymerization.

Identical treatment was also performed with cells prior to incubation on TCR stimulatory coverglasses to study effects on TCR induced actin dynamics (Fig. 8). Expectedly, disruption of F-actin by CytD or LatB fully prevented the formation of circumferential actin rings in all cases. Drug treated cells resembled those expressing Nef, however combined Nef expression and drug treatment caused even more pronounced disruption of F-actin structures. In contrast, despite efficient disruption of microtubules (Fig. 8B), nocodazole did not affect actin ring formation and cell spreading of control cells during the investigated time period and had no effect on Nef's ability to interfere with both processes. Thus, TCR induced actin dynamics and the Nef mediated inhibition thereof occur in a microtubule independent manner. Inhibition of Lck activity prevented the development of mature TCR stimulatory contacts also in the absence of Nef. However, while no actin rings were developed, cells treated with the Lck inhibitor produced actin rich, filopodia-like protrusions and membrane ruffles following contact with stimulatory surfaces (Fig. 8A). Of note, Nef potently inhibited the formation of these cell protrusions, thus efficiently interfering with the residual actin dynamics observed upon Lck inhibition. Together these results indicate an essential role of Lck in TCR coordinated actin dynamics but suggest that its kinase activity is dispensable for the inhibitory activity Nef has on these processes.

### Effects of selective Nef variants on TCR induced tyrosine phosphorylation

**Figure 9:**
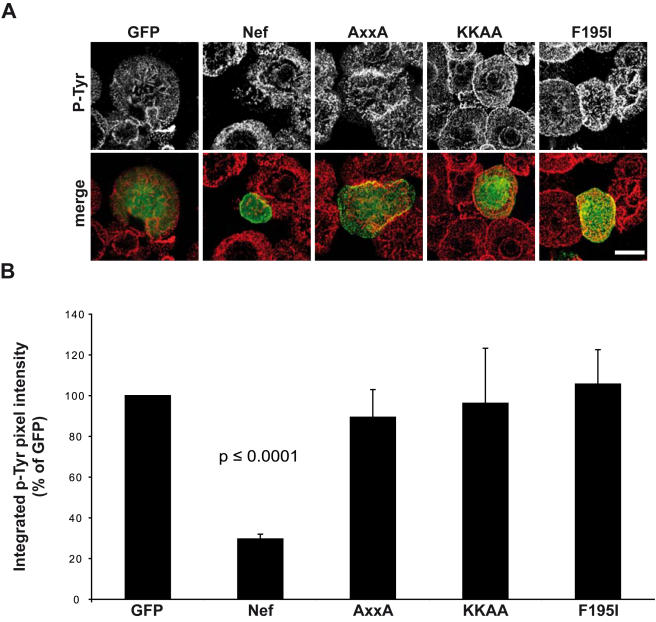
Correlation between Nef mediated inhibition of TCR induced actin dynamics and tyrosine phosphorylation. Jurkat T cells expressing GFP or the indicated Nef.GFP proteins were incubated on TCR stimulatory surfaces, fixed and analyzed for the subcellular distribution of p-Tyr positive signaling complexes by confocal microscopy. (A) Distribution of p-Tyr and GFP 5min after TCR stimulation. Results depict images representative for at least three independent experiments. White bar = 10 µM. (B) Quantification of overall p-Tyr levels of the transfected cells shown in A. Values represent the mean of three independent experiments+s.e.m. from at least 10 cells representing the phenotypes depicted in (A).

Modulation of IS architecture and function is paralleled by a reduction in TCR signal transmission that can be illustrated by measuring the induction of overall tyrosine phosphorylation early after TCR stimulation. To assess which of the different Nef activities at the IS governs this modulation in signal transmission, the discriminatory Nef mutants were used and total phosphotyrosine (p-Tyr) levels were determined by pixel quantification of confocal z-stacks (Fig. 9). Nef.GFP reduced by more than 3-fold the levels of phosphotyrosine induced upon TCR engagement relative to control cells. In contrast, the AxxA, KKAA and F195I mutant Nef proteins were all defective in this activity. These results reveal a correlation between inhibition of TCR induced actin dynamics and reduced signal transmission by Nef while intracellular Lck accumulation is dispensable for this effect.

## Discussion

The HIV-1 pathogenicity factor Nef affects several processes that are triggered upon engagement of the TCR, including actin remodeling and recruitment of Lck to stimulatory contacts. Additionally, the steady state subcellular localization of Lck is significantly changed in T lymphocytes. This study addressed whether these various activities of Nef are linked or mediated independently of each other and assessed their relative contribution to the reduction of exogenously triggered TCR signal transmission observed in Nef expressing T lymphocytes. Mapping experiments revealed that the same determinants in Nef govern its ability to prevent TCR induced actin remodeling, IS recruitment of Lck and reduction of TCR signal transmission. Surprisingly, the pronounced intracellular accumulation of Lck did not depend on some of these protein interaction surfaces in Nef and thus most likely represents an independent activity that is not directly involved in the modulation of early TCR signaling strength (see Table 1). In line with this scenario, Nef interferes with TCR induced actin remodeling in a microtubule independent manner, while sustained intracellular accumulation of Lck depends on microtubule integrity. Together, these results demonstrate that Nef has evolved independent mechanisms to modulate IS function and that the reduction of early TCR transmission is primarily mediated by interference of Nef with actin remodeling and protein recruitment to the IS.

**Table 1:** Summary of Nef effects analyzed in this study. +, full activity; +/-, intermediate activity; -, no activity; n.d., not determined

	Pak2 association	Inhibition of TCR induced actin remodeling	Intracellular Lck accumulation	Block of Lck IS recruitment	Block of TCR induced pTyr induction
Nef	**+**	**+**	**+**	**+**	**+**
G2A	**-**	**-**	**-**	n.d.	n.d.
AxxA	**-**	**-**	**-**	-	-
E4A4	**+/-**	**+/-**	**+/-**	n.d.	n.d.
EDAA	**+**	**+**	**+**	n.d.	n.d.
KKAA	**+/-**	**-**	**+**	**-**	**-**
Δ12-39	**+/-**	**+/-**	**+**	n.d.	n.d.
F195A	**-**	**-**	**+**	n.d.	n.d.
F195I	**-**	**-**	**+**	**-**	**-**

The mapping analysis performed herein revealed that Nef's effects on actin remodeling and IS recruitment of Lck are governed by identical molecular determinants. Importantly, these two activities of Nef at the IS correlated with reduced induction of tyrosine phosphorylation following TCR engagement, suggesting that these two alterations in IS organization induced by Nef are mechanistically linked to reduce early TCR signal transmission. The hierarchy between these events is not entirely clear, however, the inability of Nef expressing cells to adequately remodel their actin cytoskeleton in response to exogenous TCR stimulation appears to be imprinted already prior to reception of the stimulus [13]. This lack in actin dynamics could therefore conceivably cause a subsequent reduction in Lck translocation towards the IS. In line with such a scenario, IS recruitment of Lck is coordinated by CD4 and CD28, that induce Lck accumulation at the IS in membrane microdomains in an actin dependent manner [18], [34], [35], [36], [37]. Localized Lck activity also contributes to TCR induced actin remodeling [38]. Thus, reduced IS recruitment of the kinase in the presence of Nef may further emphasize the defects in actin rearrangements. Since disruption of actin dynamics and Lck recruitment required the microdomain association of Nef, future studies will address if actin dependent microdomain and receptor clustering constitutes the earliest event following TCR engagement that is affected by Nef. Independently of the detailed mechanism, the reduction in Lck recruitment to sites of TCR engagement correlates well with the diminished tyrosine phosphorylation and is thus likely instrumental for defects in TCR signal transmission in the presence of Nef.

One surprising finding of this study was that intracellular Lck accumulation and IS recruitment are regulated via distinct protein interaction surface of Nef. While both activities require an intact SH3 binding motif in Nef, the F195A Nef mutant revealed the uncoupling of Lck accumulation from Nef-Pak2 association and actin remodeling. The mapping to a Pak2 independent activity of the PxxP motif is reminiscent of the Nef mediated reduction of cell surface exposure of MHC-I and chemokine receptor molecules [28], [39], [40], [41], [42], [43]. Although the precise identification of these intracellular compartments warrants a refined analysis by electron microscopy, Nef co-localizes with these receptors in perinuclear compartments that might be, at least in parts, identical to the RE-like compartment of Lck accumulation. However, donwmodulation of these receptors also requires the E4 acidic cluster that is dispensable for the intracellular accumulation of Lck [26], [40], [44]. This might reflect the need for binding to the PACS sorting adaptor via this motif in order to target the receptors to this compartment. Lck in contrast is already present at the compartment, with Nef emphasizing this localization. As minimal amounts of Nef were sufficient to trigger massive accumulation and the interaction surface of the Lck containing NAKC complex was dispensable for this activity, a direct interaction between Nef and Lck is likely not required for this effect. We rather favor the hypothesis that Nef, via the PxxP motif, generally affects transport at the level of early and recycling endosomes. Such a mechanism has already been proposed for transmembrane proteins such as transferrin receptor [45], a model that is now extending to membrane associated proteins and possibly lipids. One important aspect of future studies will therefore be to address how global these effects of Nef on the RE-like compartment are and by which mechanism Nef is affecting its activity. Given the magnitude of this effect and its conservation among lentiviral Nef proteins, it will also be of interest to understand which aspects of T lymphocyte biology are specifically affected by this Nef induced relocalization. This will require the identification of Nef determinants that specifically mediate this activity. Together, this study provides first evidence that Nef affects dynamic aspects of the IS upon TCR engagement at the levels of endosomes and the plasma membrane via distinct molecular mechanisms. The IS therefore emerges as a model system that allows to study Nef's effects on protein sorting and signal transduction in parallel on a single cell level. Understanding how Nef coordinates these activities to manipulate IS function will likely provide important clues on how the viral protein boosts virus replication in the infected host.

## Materials and Methods

### Cells, reagents and plasmids

Jurkat TAg cells were cultivated in RPMI 1640 supplemented with 10% fetal calf serum, 1% L-glutamine and 1% penicillin-streptomycin (all from Invitrogen). For T cell spreading, the monoclonal antibody (mab) anti-CD3 (clone HIT3a) was used (BD Pharmingen). Further analyses were performed with the following antibodies: rabbit anti-phospho-tyrosine (BD Pharmingen), mab anti-Lck (clone 3A5) (Santa Cruz), mab anti-α-Tubulin (clone B-5-1-2), and mab anti-GFP (clone GFP-20, Sigma). Polyclonal rabbit serum against GFP was kindly provided by Hans-Georg Kräusslich. The secondary goat anti-mouse and anti-rabbit Alexa Fluor 568 antibodies were purchased from Invitrogen, the protease inhibitor cocktail was purchased from Sigma. For F-actin stain, tetramethylrhodamine isothiocyanate (TRITC)-conjugated (Sigma) phalloidin was used. The following inhibitors were obtained from Calbiochem: Cytochalasin D, Latrunculin, Nocodazole, and Lck inhibitor (4-Amino-5-(4-phenoxyphenyl)-7H-pyrrolo[3,2-d]pyrimidin-7-yl-cyclopentane). Expression constructs for Nef.GFP and Nef.RFP proteins as well as the bicistronic Nef expression constructs were described elsewhere [13], [22], [29]. Nef from HIV-1 SF2 was used throughout if not indicated otherwise. Constructs for expression of Nef.GFP and Nef.RFP carrying F195A or F195I mutations, respectively, were generated by site-directed mutagenesis (QuickChange Site-Directed Mutagenesis Kit, Stratagene) and the Nef coding sequences were verified by sequencing.

### Western Blotting

For Western blot analysis, samples were boiled in SDS Sample Buffer, separated by 10% SDS-PAGE and transferred to a nitrocellulose membrane. Protein detection was performed following incubation with appropriate first and secondary antibodies using the super signal pico and femto detection kit (Pierce, Bonn, Germany) according to the manufactureŕs instructions.

### *In vitro* kinase assay (IVKA)

Nef associated Pak2 activity was analyzed in an *in vitro* kinase reaction following immunoprecipitation of Nef.GFP essentially as described [22], [29]. Jurkat TAg cells were transfected with the respective plasmids via electroporation and incubated for 24 hours. Cells were lysed in KEB (137 mM NaCl, 50 mM Tris HCl (pH 8), 2 mM EDTA, 0.5% Nonidet P-40, Na_3_VO_4_ and protease inhibitors) and subjected to immunoprecipitation using rabbit anti-GFP serum as described. After extensive washing in KEB, beads were resuspended in 50 µl KAB (50 mM HEPES pH 8, 150 mM NaCl, 5 mM EDTA, 10 mM MgCl_2,_ 0.02% Tx100). Addition of 10 µCi [γ-^32^P] (5min, RT) ATP allowed the detection of Pak2 autophosphorylation. Following extensive washing in KEB, IVKA reactions were separated by SDS-PAGE and blotted on a nitrocellulose membrane. Radioactive signals were visualized and quantified by Phosphoimager (Bio-Rad). Immunoisolated proteins were detected by Western analysis using anti-GFP antibodies and quantified using the QuantityOne software (Bio-Rad). Radioactive signals were normalized against the amount of isolated Nef in the Western blot and signals of Nef.GFP were arbitrarily set to 100%.

### Immunofluorescence analysis

5×10^6^ Jurkat TAg cells were transfected with 15–20 µg total plasmid DNA via electroporation (960 µF, 250V, Biorad Genepulser). Microscope coverglasses (Marienfeld) were prepared by incubation in 0.01% Poly-L-lysine (Sigma) solution for 45min at 37°C and subsequent air drying. 24 hours post-transfection, 3×10^5^ Jurkat cells in 50 µl medium without additives were plated on the coverglasses, incubated for 5min and subsequently fixed for 10 min by directly adding 3% paraformaldehyde (PFA). After permeabilization with PBS/0.1% Tx-100 for 1 min, cells were blocked with PBS/1% bovine serum albumin (BSA) for 30 min. Indirect immunofluorescence was performed by incubating cells with 1∶50 (anti-Lck) and 1∶100 (anti-phospho-tyrosine) diluted primary antibodies for 3 hours and overnight, respectively. After washing with PBS, fluorochrome labelled secondary antibodies (1∶2000) were added for 1 hour. The anti-phospho-tyrosine stain was performed by using TBS instead of PBS. For F-actin staining, cells were treated with TRITC-conjugated phalloidin (1∶1000) in combination with the secondary antibodies or directly after the blocking step. For the anti-tubulin stain, cells were fixed and permeabilized with PHEMO fixation and buffer solution as described by Dohner et al. [46]. Coverglasses were mounted in Histogel (Linaris) and analyzed with a LSM 510 confocal laser scanning microscope (Zeiss). Images were taken using a 100×oil immersion objective and processed using Adobe Photoshop.

### Analysis of TCR mediated actin ring formation

T cell spreading on stimulatory surfaces was performed essentially as described [47]. Briefly, microscope coverglasses were cleaned with 1M HCl/70% ethanol for 30min and dried at 60°C for 30 min before treating with a 0.01% Poly-L-lysine solution for 10min. For antibody coating, dried cover glasses were then covered with anti-CD3 antibody diluted in PBS (7–10 µg/ml) for 3 hours at 37°C. After washing with PBS, the coverglasses were stored in PBS at 4°C. Cells were added in a volume of 50 µl onto the glasses, incubated for 5min at 37°C and fixed by direct addition of PFA. Further steps were performed as described above.

### Intensity measurements and 3D-reconstructions

Lck and phospho-tyrosine levels of transiently transfected Jurkat cells were analyzed by determination of the integrated pixel intensity of the immunofluorescence preparations with ImageJ software. Therefore, serial confocal sections of x-y images from individual cells along the z-axis with the distance of 1 µm were obtained scanning a representative number of cells from top to bottom. The total signal of each cell was determined by summarizing the integrated densities of the single sections of from the entire z-stack. Contact site recruitment of the respective signal in individual cells was determined by comparing the integrated pixel intensity of the confocal section representing the surface contact with the total integrated pixel intensity. Statistical analyses were performed with the Student's *t*-test. 3D-reconstructions of confocal stacks were performed with the stacks-Z-function plugin of ImageJ and with the 3D-application of Zeiss LSM software.

### Drug incubation

For pharmacological inhibition of the actin and tubulin cytoskeleton and Lck activity, transiently transfected Jurkat T cells were treated with either 5 µM cytochalasin D, 0.5 µM latrunculin, 3 µM Lck inhibitor or 10 µM nocodoazole in medium for 30min. After plating the cells in medium still containing the inhibitor for 5min on coverglasses, cells were fixed and prepared for immunofluorescence analysis as described above.
